# Estimating data-driven coronavirus disease 2019 mitigation strategies for safe university reopening

**DOI:** 10.1098/rsif.2021.0920

**Published:** 2022-03-14

**Authors:** Qihui Yang, Don M. Gruenbacher, Caterina M. Scoglio

**Affiliations:** Department of Electrical and Computer Engineering, Kansas State University, Manhattan, KS 66506, USA

**Keywords:** COVID-19, SARS-CoV-2, agent-based model, non-pharmaceutical interventions, social contact, vaccination

## Abstract

After one pandemic year of remote or hybrid instructional modes, universities struggled with plans for an in-person autumn (fall) semester in 2021. To help inform university reopening policies, we collected survey data on social contact patterns and developed an agent-based model to simulate the spread of severe acute respiratory syndrome coronavirus 2 in university settings. Considering a reproduction number of *R*_0_ = 3 and 70% immunization effectiveness, we estimated that at least 80% of the university population immunized through natural infection or vaccination is needed for safe university reopening with relaxed non-pharmaceutical interventions (NPIs). By contrast, at least 60% of the university population immunized through natural infection or vaccination is needed for safe university reopening when NPIs are adopted. Nevertheless, attention needs to be paid to large-gathering events that could lead to infection size spikes. At an immunization coverage of 70%, continuing NPIs, such as wearing masks, could lead to a 78.39% reduction in the maximum cumulative infections and a 67.59% reduction in the median cumulative infections. However, even though this reduction is very beneficial, there is still a possibility of non-negligible size outbreaks because the maximum cumulative infection size is equal to 1.61% of the population, which is substantial.

## 1. Introduction

Severe acute respiratory syndrome coronavirus 2 (SARS-CoV-2) outbreaks have led to unprecedented restrictions on higher education institutions worldwide. From March 2020 to August 2021, most universities in the USA suspended in-person operations and employed remote or hybrid instructional modes. During the summer months of 2021, buoyed by the wider vaccine availability, a growing number of universities were planning for an in-person autumn (fall) semester. As there would be people returning to campus through out-of-state or international travel, it was hard to project the vaccination coverage at the beginning of the coming semester. In addition, despite the high effectiveness of coronavirus disease 2019 (COVID-19) vaccines, many institutions have not required a vaccine mandate. Prior studies have provided various estimations for vaccination hesitancy and suggested that COVID vaccination hesitancy appears high in certain population subgroups such as young adults [1–4]. For example, Latkin *et al*. [1] found that 60.6% of people aged 18–29 years old were vaccine hesitant during 14–18 May 2020. Sharma *et al*. [3] reported that 47.5% of participants were hesitant to get vaccinated based on questionnaires distributed to college students at a southern US university in February and March 2021. The vaccine hesitancy was about 42% among French university students, according to a study conducted by Tavolacci *et al*. [5] in January 2021. Although vaccine hesitancy has decreased as more people are getting vaccinated [6], there are still many people who are reluctant to have a COVID-19 vaccination.

In addition, COVID vaccine immunity wanes over time. A study reported that mRNA BNT162b2 COVID-19 vaccine effectiveness against SARS-CoV-2 reduces from 88% to 47% after five months of full vaccination [7]. Such vaccine-waning effects can influence the control measures needed to achieve herd immunity [8]. Booster shots can help restore immune responses. Despite the debate surrounding COVID-19 booster shots, several countries, including the USA, have offered booster doses of COVID-19 vaccines to adults.

Facing such challenges, universities struggle with plans to resume normal operations while mitigating the risks of SARS-CoV-2. Since the pandemic started, over 700 000 cases have been reported concerning American colleges and universities [9]. Several US-oriented modelling studies have been conducted to guide decision-making on testing strategies, mask usage, social distancing and class sizes during university reopening [10–16]. Focusing on the impact of testing, contact tracing and remote courses on epidemic dynamics, Gressman & Peck [11] modelled daily interactions among a 22 500-person urban university population and divided students into groups based on course schedules. Bahl *et al*. [12] developed a detailed agent-based susceptible–exposed–infected–recovered (SEIR) model with a university population of 2380, focusing on small, closed-community residential colleges. In their model, each agent randomly moves between nodes in a graph based on a fixed hourly schedule. They simulated friend groups, but were not able to incorporate contact tracing functionality because of the model structure. Junge *et al*. [10] incorporated 16 800 agents and simulated a discrete day-by-day dynamic interaction graph. Under an average reproduction number $R_{0}=3$, they found that vaccine coverage over 80% makes it possible to resume in-person instructions safely. Owing to data unavailability, many of these models simulate contacts based on random mixing or assumptions regarding class schedules and common locations. SARS-CoV-2 transmission in universities is a global issue, and research on this topic is fast paced. Contact patterns could vary along with the university settings and geographical locations, and there are models applied to other countries, such as UK-oriented studies [17,18] and a Canada-oriented study [19]. Previous studies have shown that contact networks could significantly impact the accuracy of epidemic predictions and the effectiveness of control strategies [20–22], highlighting the importance of data collection on real-world contact patterns. Similar to the models in [10–12], each individual in our model follows a schedule to move to different locations and can have contacts with other individuals that are in the same location. The main difference between our work and previous studies is that our model is tailored in such a way as to use the social contact survey data we collected about social interactions in a US university as much as possible.

SARS-CoV-2 is still evolving, and researchers are devoted to retrieving its epidemiological parameters [23–25]. Recent studies have reported non-exponential distributions for critical transition times between different disease states, such as the infectious period [26,27]. However, most epidemic models have been developed based on Markovian processes with transition times following exponential distributions. Such unrealistic assumptions could impair the accuracy of model predictions, and non-Markovian models that accept arbitrary distributions for the transition times of the individual between different compartments have started to draw attention from scholars [28–30]. Previous studies have highlighted that agent-based modelling (ABM) has offered a practical way to incorporate details about heterogeneous contact networks, compared with other computational models. However, previous studies did not explicitly mention the advantage of ABMs over computational models with respect to non-exponential transition times. Emerging methods focus on non-Markovian models, but they are often associated with complicated derivations based on differential equations. By contrast, although often at a higher computational cost, non-exponential distribution parameters, if available, can be easily incorporated into ABMs, which is critical for epidemic forecasting.

In this study, we develop an ABM to examine the mitigation strategies needed for safe university reopening in the 2021 autumn semester. The model incorporates a social contact network based on survey data. Considering different immunization effectiveness, we simulate SARS-CoV-2 spreading in a university population under two scenarios: (i) relaxation of non-pharmaceutical interventions (NPIs) and (ii) adoption of NPIs, such as wearing masks. In addition, we perform a thorough model calibration and accurate sensitivity analyses on immunization effectiveness. The outcomes are valuable to understand the impact of initial immunity levels on the future epidemic spread, thereby helping inform university-reopening policies.

The contributions of this paper are summarized as follows:

— We develop an ABM incorporating non-Markovian transition times and real contact networks based on survey data.
— With 70% immunization effectiveness and $R_{0}=3$, we estimate that at least 80% of the university population immunized through natural infection or vaccination is needed to ensure a healthy campus with relaxed NPIs.
— We observe that the implementation of NPIs can dramatically reduce the maximum cumulative infection, and continued NPIs are recommended to mitigate risks of large-gathering events.

## 2. Survey data

To parametrize the model, we conducted a social contact survey administered to all students, faculty and staff at Kansas State University between 2 December 2020, and 25 January 2021. We sent emails to 6196 faculty and staff members and 20 755 students, and received responses from 3581 participants. The survey asked the participants to report their typical social interaction patterns during the semester. The survey data contain information about age segment, role at the university, housing status and number of close contacts categorized by duration ranges in a week. More specifically, close contacts are referred to as the people the participants meet at a distance of less than 2 m. We also gathered information about visit frequency, duration and the number of contacts at different locations.

In the electronic supplementary material, figure S1 depicts the age, housing status and the number of close contacts in a week by duration ranges. We can see that 50.70% of participants reported being in the age category 18–24, followed by 15.90% of participants in the age category 25–35. Regarding housing status, 9.82% of students live in sororities or fraternities (here, sororities refer to buildings where female undergraduates live and fraternities refer to buildings where male undergraduates live), 21.00% live in on-campus housing and the remaining students live in off-campus apartments or houses. The majority of faculty and staff (98.91%) choose off-campus housing options. In the survey, we explicitly indicate that examples of contacts who regularly meet for more than 4 hours per week are roommates, family members or co-workers. Contacts between 1 and 4 h per week may refer to friends or classmates, and contacts between 15 min and 1 h per week could be friends or others that the participant might occasionally meet. Overall, the contact patterns categorized by role reveal that students living in sororities or fraternities have more contacts while faculty and staff have fewer contacts. For example, regarding the duration of more than 4 h per week (electronic supplementary material, figure S1, yellow), the median number of contacts for students living in sororities or fraternities is eight contacts compared with faculty and staff with two contacts. More statistics about the survey data can be found in the electronic supplementary material.

## 3. Model

The model developed mainly consists of two types of agents, namely person and location agents. During initialization, we create 26 000 person agents, representing 20 000 students and 6000 faculty and staff. Each person agent is assigned to one role category of (i) faculty and staff, (ii) student living on-campus, (iii) student living off-campus, or (iv) student living in a sorority or fraternity. The age distributions of these four role categories obtained from the survey data are detailed in the electronic supplementary material, table S1, which shows that age distributions vary among the role categories. For example, 98.73% of students living on-campus are aged 18–24, while 72.66% and 19.28% of students living off-campus are aged 18–24 and 25–35, respectively. Accordingly, we used these four age distributions to characterize the agents in the corresponding role category, instead of using the age distribution over the whole campus population. Each location agent plays one role of (i) recreation centre or any gym or other shared exercise spaces; (ii) union, dining centres and coffee shops on-campus; (iii) bars, restaurants and coffee shops off-campus; (iv) stores and other types of services off-campus; and (v) other types of social gathering such as sport, religious and social events.

### 3.1. Daily activities

**Figure 1.**
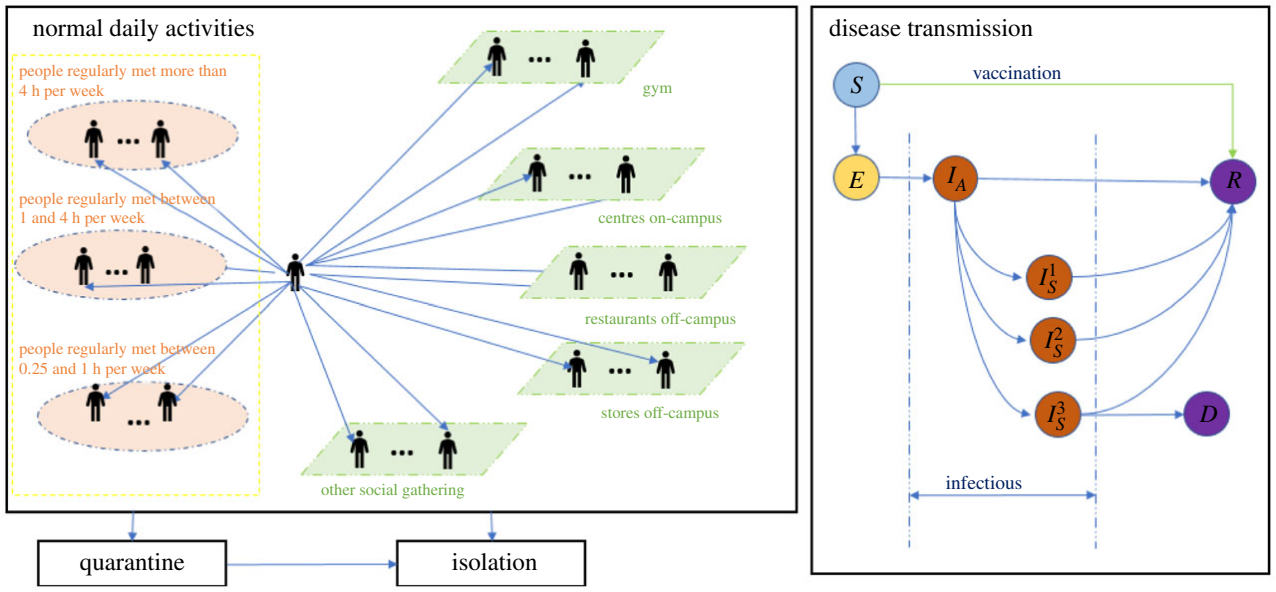
Structure of the person agent in the model.

Figure 1 depicts the structure of the person agent. Each person agent has contact with people it regularly meets in the three contact lists and meets other people at the five types of locations. More specifically, the people within each person's contact lists are randomly selected from the whole population during model initialization and remain unchanged throughout each simulation run. By contrast, the people each person meets at specific locations are dynamic, as the contact will only occur if two agents are within the same location. At different times of the day, each person agent visits these locations based on the visit frequency, the duration and the number of contacts, which are sampled from custom distributions in AnyLogic defined using the survey data. The frequency of a value sampled is dependent upon the number of times this value occurs in the survey data.

### 3.2. Disease transmission

In line with [31,32], each individual has a state reflecting its health status: susceptible (*S*), exposed (*E*, infected but not infectious), asymptomatic $\left(I_{A}\right)$, symptomatic differentiated by mild $\left(I_{s}^{1}\right)$, severe $\left(I_{s}^{2}\right)$ and critical illness $\left(I_{s}^{3}\right)$, recovered (*R*) and dead (*D*). Contacting an infectious individual, a susceptible individual could become infected and transition to the exposed state based on the transmission probability per contact β and risk factors of the contacts. An individual in the exposed state will transition to the infectious state *I_A_* after a period lognormal distributed with a mean of 4.5 days and a standard deviation of 1.5 days [33–36]. Infected individuals may develop symptoms based on age-dependent probabilities [37,38]. The probabilities that symptomatic cases develop into mild, severe or critical illness are also age dependent [38]. The length of time for an individual to transition from state *I_A_* to symptom onset ($I_{s}^{1}$, $I_{s}^{2}$ or $I_{s}^{3}$) follows a lognormal distribution with a mean of 1.1 day and standard deviation of 0.9 days [25,27]. The recovery time for asymptomatic cases and mild symptomatic cases is a period sampled from a lognormal distribution with a mean of 8 days and a standard deviation of 2 days [39]. Individuals with severe and critical illness recover after a period sampled from a lognormal distribution with a mean of 18.1 days and a standard deviation of 6.3 days [37]. Accounting for outside transmissions due to non-university contacts, we assume the university population has few contacts with local communities, and more details can be found in the electronic supplementary material.

### 3.3. Testing, quarantine and isolation

When an individual has mild symptoms (state $I_{s}^{1}$), it may get a SARS-CoV-2 test based on a 70% probability [12]. Individuals in the critical or moderate symptomatic state ($I_{s}^{2}$ or $I_{s}^{3}$) may get tested with a 95% probability [12]. The delay for returning test results is uniformly distributed between 0 and 2 days. An individual will be isolated for 10 days after testing positive. If the individual shows symptoms, the time spent in isolation will count starting from its first day showing symptoms. In addition, a percentage of contacts of a positive case can be identified via contact tracing and be quarantined after a delay uniformly distributed between 0 and 3 days [40]. During the quarantine period, the agents may get tested after showing symptoms based on the above probabilities and will be isolated once they test positive. The agent will stop daily activities in both quarantine and isolation states.

### 3.4. Relaxation and adoption of non-pharmaceutical interventions

As reported in [41–43], NPIs such as wearing masks could reduce the infection likelihood by at least 50%. Consider that there was a mask mandate in all the on-campus facilities, and there was local guidance about wearing masks in public spaces out of the campus during the autumn semester of 2020. When NPIs are implemented, we keep the risk factor to be 1 for close contacts (people regularly meet for more than 4 h), simulating that transmission probability for contacts with roommates, family members and co-workers is unchanged. In addition, we set the risk factor to be 0.5 for all the other types of contact to reduce the transmission probability by half.

### 3.5. Model calibration

To estimate the unknown parameters, including the transmission probability per contact β, the contact tracing percentage and the initial number of people in the exposed state, we use the simulation-based optimization method provided in AnyLogic software. The OptQuest Engine, which incorporates metaheuristics to guide its search algorithm towards better solutions [44], is used as an optimization engine to minimize the difference between the cumulative confirmed cases from the simulation and the reported positive cases that are published weekly in the university dashboard from 17 August 2020 to 30 October 2020. During this period, mask usage was mandated to campus and public spaces in local communities. In the calibration process, the transmission probability per contact β is set to change every 7 days. In the electronic supplementary material, figure S4 shows the number of cumulative confirmed cases compared with the historical data. The sum of the absolute deviation of the outputs is 117.5, calculated from $S=\underset{s}{\sum }|o_{s}-y_{s}|$, where $o_{s}$ and $y_{s}$ are the median cumulative confirmed cases and historical data from the dashboard. The simulated median generation time is 5.96 days, with an interquartile range of 4.58–7.98 days. With all other agents in the susceptible state and not considering control measures, we provide empirical measures of $R_{0}$ from the simulations, which is computed as the number of secondary infections directly associated with the initial exposed cases divided by the number of initial exposed cases. We find that an infection transmission probability per contact of 0.05 would return a median reproduction number of $R_{0}=3$.

### 3.6. Assumptions for estimating the herd immunization threshold

We consider a two-dose vaccination allocated to susceptible people with a time interval of 28 days. The vaccination roll-out rate is uniformly distributed between 0 and 10 doses per day. Vaccine efficacy is modelled as the probability that a vaccinated susceptible agent directly transitions to the recovered state. Denote the vaccine efficacy of the first dose as *V_e_*, and the vaccine efficacy of the second dose is calculated as $V_{e}\cdot 0.956/0.92$, based on the report [45] in which vaccine efficacy is 92% for the first dose and 95.6% for the second dose. To initialize the epidemics, we randomly select 30 individuals from the university population, and the selected susceptible individuals will transition to the exposed state. During scenario analyses, the initial immunization coverage *α* is the percentage of the university population that has already had prior infection or has been vaccinated by the start of the semester. At the start of the simulation, the *α* percentage of the population will be selected and set to be in the recovered state based on a probability, which will be referred to as immunization effectiveness hereafter. During the simulation period, people can be vaccinated and transition in the recovered state based on the same probability, namely the immunization effectiveness. Susceptible individuals could get infected and eventually become recovered. Note that once individuals are marked as recovered, they will remain in the recovered state throughout the simulation run. We perform 1000 simulation runs for each scenario. In each simulation run, we record the number of active cases and cumulative infected cases on a daily basis.

## 4. Results and discussions

In this section, we present and compare simulation results under scenarios of NPI relaxation (wearing masks and maintaining social distancing are optional) and NPI adoption (wearing masks and maintaining social distancing are mandatory). In each scenario, we vary the initial immunization coverage, α, which refers to the percentage of people immunized through natural infection or vaccination at the start of the autumn semester.

### 4.1. Non-pharmaceutical intervention relaxation

**Figure 2.**
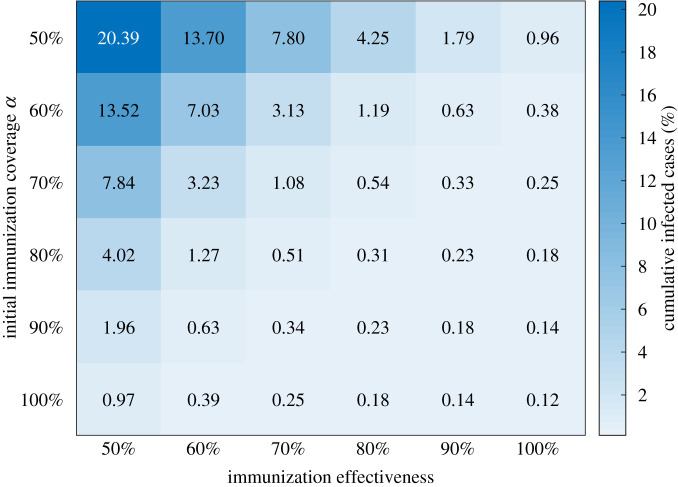
Heat map showing cumulative infected cases as a function of immunization effectiveness and initial immunization coverage α under relaxed NPIs and *R*_0_ = 3. The colour code represents the median percentage of cumulative infected cases by the end of the semester. The number of simulation runs is 1000.

Figure 2 shows the heatmap with median percentages for the cumulative infections by the end of the semester with *R*_0_ = 3. We can see that the initial immunity level α needed for a safe university reopening is sensitive to the changes in the immunization effectiveness. With 70% immunization effectiveness, α=60%  and α=80%  can lead to 3.13% (1.34–5.09%) and 0.51% (0.36–0.85%) cumulative infections of the university population, respectively. With 90% immunization effectiveness, 60% initial coverage can result in cumulative infections in 0.63% (0.38–1.04%) of the population, and disease outbreaks can be controlled relatively well.

**Figure 3.**
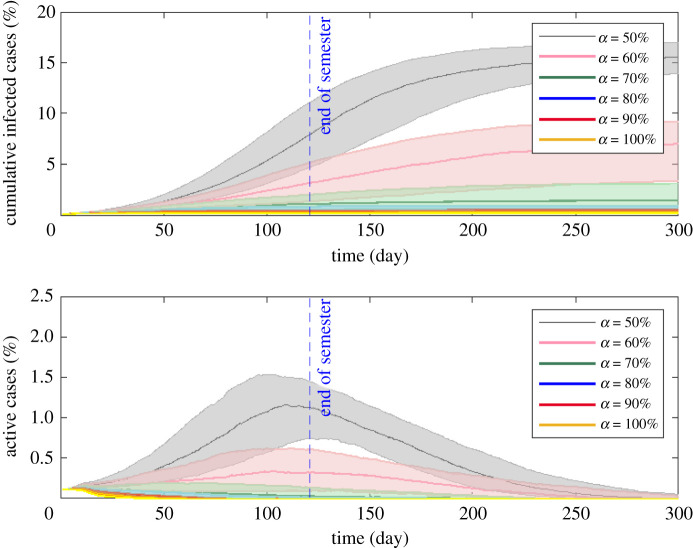
Effect of initial immunization coverage *α* on cumulative infected cases and active cases with 70% immunization effectiveness and *R*_0_ = 3 under relaxed NPIs. The beginning of the simulation corresponds to the start of the semester, and the vertical dashed blue line indicates the end of the autumn semester. While our main focus is to examine the epidemic dynamics over the coming autumn semester, we provide a relatively complete picture of the epidemic curve up to day 300. The initial immunization coverage *α* is varied between 50% and 100%, with an interval of 10%. The solid lines show the median percentages of cumulative infected and active cases, and the shaded areas represent the 25th percentile and 75th percentile interquartile range of 1000 simulation runs.

In the following, we provide more simulation results with 70% immunization effectiveness. Considering relaxed NPIs, figure 3 shows the median percentage of cumulative infections and active cases over time given different initial immunization coverages. While our main focus is to examine the epidemic dynamics over the autumn semester, we depict the simulation results up to day 300 to provide a relatively complete picture of the epidemic curve. A summary of the statistics is listed in table 1. When *α* increases from 50% to 80%, the median value for the peak of infection reduces dramatically from 1.57% to 0.15% of the total population and slightly declines after *α* = 80%. When *α* = 80%, the peak occurs at day 11 with an interquartile range of 7–23.50, and the epidemic is well contained. The percentage of cumulative infections by the end of the semester decreases dramatically from 7.80% (interquartile range: 4.62–10.98%) for α=50%  to 0.51% (interquartile range: 0.36–0.85%) for α=80% . At the time of the original writing (July 2021), vaccination rates in some US states remain low. For example, only 36.0% of the state's population is fully vaccinated in Arkansas. The simulation results show that such a relatively low vaccination rate could lead to a large number of infections under relaxed NPIs.

**Table 1.** Summary statistics. Peak time is the number of days elapsed after the initial infection. For each simulation run, we calculate the peak value and peak time of active cases. In the table, median values are associated with interquartile ranges. The attack rate is calculated as cumulative infections/ (total population×(1−α×immunization effectiveness)).

initial immunity level *α*	peak value of active cases (%)	peak time (days) of active cases	cumulative infections (%)	attack rate (%)
50%	1.57 (1.25–1.93)	120 (96–152.5)	15.53 (13.92–17.02)	23.90 (21.42–26.18)
60%	0.65 (0.31–0.92)	102 (65–148)	7.01 (3.37–9.23)	12.08 (5.82–15.91)
70%	0.21 (0.15–0.35)	35 (11–84)	1.42 (0.66–3.12)	2.79 (1.30–6.12)
80%	0.15 (0.13–0.18)	11 (7–23.5)	0.56 (0.39–0.93)	1.27 (0.89–2.11)
90%	0.13 (0.12–0.15)	8 (6–11)	0.37 (0.50–0.28)	1.00 (0.77–1.36)
100%	0.13 (0.12–0.13)	6 (5–9)	0.26 (0.22–0.32)	0.86 (0.72–1.08)

**Figure 4.**
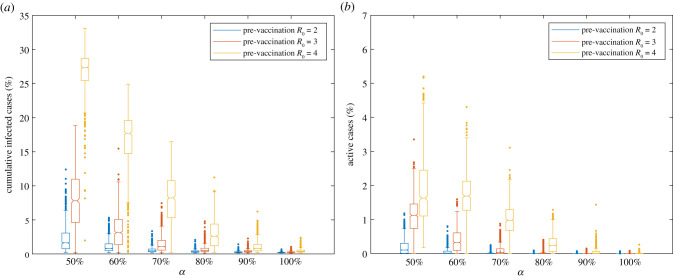
The impact of pre-vaccination *R*_0_ on cumulative infected cases and active cases under the relaxation of NPIs and 70% immunization effectiveness. The number of simulation runs for each scenario is 1000.

As a piece of additional information, we show the impact of $R_{0}$ on cumulative infections and active cases in figure 4. Immunization coverage *α* = 60% and *α* = 90% can ensure a relatively safe reopening for $R_{0}=2$ and 4, respectively. As expected, the immunization threshold is largely dependent on the value of $R_{0}$, indicating the importance of an accurate estimate for $R_{0}$.

### 4.2. Non-pharmaceutical intervention adoption

**Figure 5.**
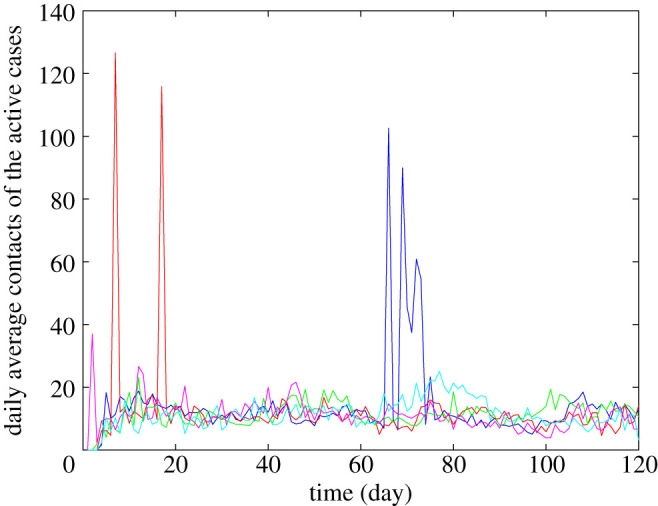
Average daily contacts of the active cases. The figure depicts the average daily contacts of the active cases for five simulation runs (blue, red, green, magenta and cyan) with the top five cumulative infections among all 1000 simulation runs. The simulation considers relaxed NPIs with 70% immunization effectiveness, *α* = 80% and *R*_0_ = 3.

Figure 5 plots the average daily contacts of infectious people. Since social interactions among the agents are based on empirical distributions, we observe that a high number of contacts occasionally could occur in certain simulation runs owing to the stochasticity of the simulations, potentially leading to a large number of infections. We refer to this phenomenon as large-gathering events, implying large gatherings at which an infectious person could be in contact with many people.

**Figure 6.**
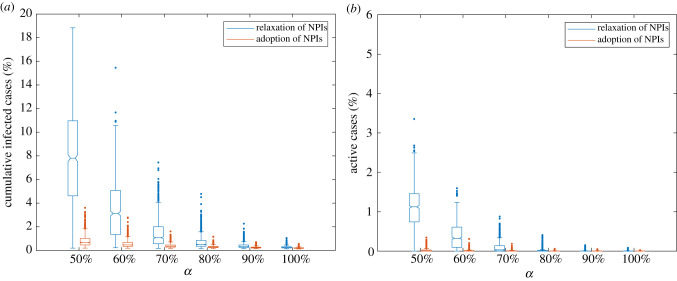
Distributions of the cumulative infected cases and active cases in the percentage of the total population by the end of the semester. The figure shows results under the relaxation of NPIs and the adoption of NPIs considering 70% vaccine effectiveness and *R*_0_ = 3. The number of simulation runs for each scenario is 1000.

Figure 6 shows the impact of NPIs on the cumulative infected cases and active cases given different initial immunization coverages. When NPIs are relaxed, the median percentage of active cases at the end of the semester for *α* = 70% is 0.03%, but there are multiple outliers of over 0.50% a day. Similarly, the cumulative infections at *α* = 70% have median values of 1.08%, but have multiple outliers over 5.00% of the university population. For *α* = 80%, at which the median value for the active cases equals 0, the maximum cumulative infected cases can reach up to 4.78% of the population. Although such undesired outcomes rarely happen, this suggests that large-gathering events might result in large outbreaks.

In comparison, we observe that continuing NPIs can cause a dramatic reduction in the epidemic size and can substantially reduce the risks of large outbreaks. When NPIs are adopted, the median percentages of cumulative infected cases range from 0.20% to 0.28% for α equalling 80–100%, and ranges from 0.35% to 0.68% when α falls below 80%. In addition, compared with scenarios with relaxed NPIs, continuing NPIs can substantially reduce risks of large outbreaks. For instance, the number of maximum cumulative infected cases reduces from 7.45% with the relaxation of NPIs to 1.61% with the adoption of NPIs for α=70%.

## 5. Conclusion

As the autumn semester was approaching, many universities had announced the plan for an in-person semester, with relaxed SARS-CoV-2-related guidelines. Without a vaccination mandate policy, the exact immunity level of the university population was largely unknown, considering that many students, faculty and staff were coming back through out-of-state or international travel. Our simulation results suggest that, on average, an immunization coverage of 80% of the university population could lead to safe university reopening with relaxed NPIs, $R_{0}=3$ and 70% immunization effectiveness (figure 4). However, an immunization coverage below 80% may pose significant risks to the public health of the university population with the relaxation of NPIs, and attention needs to be paid to the large-gathering events that may lead to large infections.

Since the SARS-CoV-2 outbreak, studies have shown that encouraging people to get vaccinated and continuing non-pharmaceutical control policies are effective ways to suppress the disease spread [46–48]. Consistently, we conclude that it is possible to ensure a healthy campus community associated with NPIs at lower immunization coverages. At the same initial immunization coverage of 70%, adoption of NPIs could lead to a 78.39% reduction concerning the maximum cumulative infections (figure 6), which reflects the possible non-negligible infection from large-gathering events. Therefore, it is recommended that people continue to exercise social distancing measures for the coming autumn semester.

In this study, we developed an agent-based disease transmission model based on a real contact network structure and non-Markovian transition time distributions. The contact network is based on survey data that are specific to the university population. However, selection bias may occur in our study for students who do not frequently use their university emails. For the respondents included in the analysis, only 62.50% are students, which is smaller than the actual percentage, i.e. 77.01% of the university population. This may happen because our surveys are distributed merely through emails, and faculty and staff may more frequently engage in communications through emails. In addition, the distribution date of the survey questions is during the end of the semester, and those students who have already gone back home may no longer pay attention to their university emails. This selection bias could result in a lower estimation of the immunity threshold as we observe, on average, students have more contacts than faculty and staff. Although we explicitly mentioned in each of the survey questions that the contacts of interest are those for a typical semester and the number of people the participants met at a distance of less than 2 m, inaccurate estimations may exist during their recall or misinterpretation of the definition. We draw our conclusions based on the early estimations of transmission probabilities during the pandemic. Sustaining NPIs will become even more critically important, given the highly contagious SARS-CoV-2 variants and waning immunity, which could lead to worse scenarios. One limitation of our model lies in the difficulty of setting the parameters owing to data unavailability. Therefore, we make several assumptions and conduct more simulations, the results of which can be found in the electronic supplementary material. We find that the number of infections used to initialize the outbreak and vaccination roll-out rate has only a minimal impact on the initial immunization coverage needed for a safe reopening. Another limitation is that we were not able to include the impact of holidays on contact patterns as we only include the university population in the model, and students could go to multiple places during the Thanksgiving (November) break. Nonetheless, we believe that the outcome of this study provides important messages for universities planning for a full reopening, especially those located in the states with low vaccination rates.

## Data Availability

Supporting materials and code for the simulations have been uploaded as part of the electronic supplementary material. The data are provided in the electronic supplementary material [49].
